# Associated factors of pregnancy spacing among women of reproductive age Group in South of Iran: cross-sectional study

**DOI:** 10.1186/s12884-020-03250-x

**Published:** 2020-09-22

**Authors:** Tania Dehesh, Elaheh Salarpour, Neda Malekmohammadi, Sepideh Arjomand Kermani

**Affiliations:** 1 Department of Biostatistics and Epidemiology, School of Public Health, Kerman University of Medical Sciences, Kerman, Iran; 2 Student Research Committee, School of Medicine, Kerman University of Medical Sciences, Kerman, Iran

**Keywords:** Pregnancy spacing, Abortion, Stillbirth, Breast feeding, Contraception, Recurrent event survival analysis, Iran, Kerman

## Abstract

**Background:**

Optimal pregnancy spacing is an important incidence in reproductive women’s health. Short or long pregnancy spacing leads to the greatest health, social and economic problems such as increase in maternal and infant mortality and morbidity, and adverse pregnancy outcomes. The aim of this study is to assess the mean of pregnancy spacing and associated factors of pregnancy spacing among women of reproductive age group with recurrent event analysis.

**Methods:**

The fertility history of 1350 women aged 15–49 years was collected in this cross-sectional study. The women were selected through multistage random sampling method from a list of clinics in 2018. Some predictors were collected from their records and others were collected by face-to-face interview. The recurrent event survival analysis was used to explore the effect of predictors on pregnancy spacing. The R software program was used for analysis.

**Results:**

There were nine predictors that had significant effect on pregnancy spacing. These predictors included the age of mother at marriage, mother’s BMI, contraception use, breast feeding duration of the previous child, the education level of husband, the sex preference of the mother, presence of abortion or stillbirth in the preceding pregnancies, income sufficiency, and mother’s awareness of optimum pregnancy interval. The most influential predictors; contraception use (HR = 2.34, 95%CI = 1.23 to 2.76, *P* < 0.001) and income sufficiency (HR = 2.046, 95%CI = 1.61 to 3.02, *P* = 0.018) lead to longer and son preference of mother (HR = 2.231, 95%CI = 1.24 to 2.81, *P* = 0.023) lead to shorter pregnancy spacing.

**Conclusion:**

The up to date contraception tool should be at hand for couples to manage their pregnancy intervals. The unfavorable economic situation of a family leads to long pregnancy spacing. Despite the relative equality of the status of girls and boys in today’s societies, the desire to have a son child is still an important factor in shorter pregnancy spacing. The benefit of optimal pregnancy spacing should be more announced.

## Background

Pregnancy is one of the important events in a women’s life after marriage [1]. Planning to have child has an important role in the future life of the mother, family, and society [2]. The goals of each pregnancy are healthy infants and healthy mothers. Pregnancy spacing is the time period between the current pregnancy and the preceding pregnancy [3]. Managing pregnancy spacing has important effects on mother and infant health [4]. According to World Health Organization (WHO) and other international organizations, the optimal interval between pregnancies should be 36–60 months [5]. Evidences from previous studies indicate that short and long pregnancy spacing can significantly increase the risk of adverse maternal, perinatal, infant, and child outcomes. This fact demonstrated that a pregnancy spacing that is shorter than 18 months and longer than 59 months leads to the main cause of increased risk of adverse perinatal outcomes [6]. Previous studies demonstrated that many factors such as females education [7], son preference [8], regular menstrual cycle, age of women at marriage [9] and social economic factors [10] have important effects on pregnancy spacing. Age of marriage, and woman’s education were significant predicators of the birth interval in previous study in Yazd, Iran [11]. Women age at marriage, wife and husband education, and employment status were also important predictors of pregnancy spacing in Hamadan, Iran [12]. Pregnancy is an event that may repeat in a woman. The most accurate analysis procedure for time between repeated events (pregnancy spacing in current study) is recurrent event survival analysis. According to our knowledge there is no study that investigating influential factors of pregnancy spacing with recurrent event survival analysis in Iran. In addition, in current study the effects of more predictors are investigated in analysis which their effects were not investigated in previous studies. As the social-economic situation of countries has changed, the association factors of pregnancy spacing have also changed. However, associated factors of pregnancy spacing are not well-addressed in developing countries, especially in the south of Iran, which is one of the most crowded countries in the Middle East. The aim of this study is first to determine the mean of pregnancy spacing interval and second to explore the associated factors of pregnancy spacing in Kerman, southern Iran with recurrent event survival analysis.

## Methods

In this cross-sectional study, the fertility history of 1350 pregnant women aged 15–49 years in Kerman (a well-developed city in the south of Iran) was collected from August to December 2018. The Inclusion criteria were: being pregnant at the time of interview, 15–49 years old; being resident of Kerman since marriage and Iranian nationality. Exclusion criteria: having a history of infertility and history of genital diseases. The women were pregnant at the time of interview. They came to the pregnant women consultation part of the clinics for monthly check. Some of them had medical records in clinic from previous pregnancies and their records were checked for some information. Other information was gathered by asking the women themselves. The women were selected through multistage random sampling method from a list of public health centers. In the first stage, 10 main centers were selected, then, in the second stage, 1500 samples were allocated to each centers based on the mean monthly referral of pregnant women. In fact, based on mean referral pregnant women to each clinic monthly, the total sample was divided. More samples were allocated from those clinics that covered more pregnant women. After dividing total samples among the 10 main centers, the women were randomly selected based on file numbers in each center. Data were collected using a checklist (Supplement 1 and 2) completed during face-to- face interviews carried out by three research assistants. The checklist included demographic characteristics, age at marriage, education level of wife and husband, adequacy of family income, and some other questions. These are predictors and the outcome is the time between pregnancies. The women who did not agree with the goal of this study were excluded. The woman needed to remember the history of her fertility, especially about the variable that was not recorded in the past. The imprecise answer may have caused some biases in the final result. 10% of women declined to participate in the study and were excluded. Instead of them other eligible women were included. Ethical approval was granted by the Ethics Committee of Kerman University of Medical Sciences (reference number: IR.KMU.REC.1398.078). This study was conducted in compliance with the Helsinki Declaration. All the participants gave their written informed consent and for under 16 years old women parental consent was obtained.

### Predictors

We explored predictors associated with the study outcome: age, which means the number of years from birth (years old), education status of the mother and husband (Illiterate, primary, middle school, high school, and university education), drug use status of mother and husband, which means the addict to any types of narcotics such as: opium or heroin (yes / no), income status, which means the status of monthly family income based on personal perspective. Is the family income completely sufficient for family needs according to family member’s expectations or not? (Sufficient/ insufficient), number of intercourse in month (> 2, 2–4, < 4), mother’s age at marriage which means the number of years from birth until marriage (years old), mother underlying diseases, which means existence of any chronic disease such as blood pressure (BP), kidney disease, or diabetes (yes / no), mode of delivery (vaginal / cesarean), contraception before current pregnancy (yes/no), night job of mother and husband which, means having job that must be out of home at work during night (yes / no), breast feeding of preceding child (month), mother’s BMI (BMI is a measurement of a person’s weight with respect to his or her height), and age at the first menstruation, which means the number of years from birth to the first menstruation (years old). Mother and husband’s drug use and night job of mother and husband were added to the model as confounders.

### Statistical analysis

Pregnancy is an event that could be repeated several times in a woman’s life, so the recurrent event survival analysis could be the most suitable statistical model to explore the effect of different types of predictors on pregnancy spacing. Survival analysis is used when the outcome is time until the event. In recurrent survival analysis, the time between events is called outcome. Cox proportional hazard model was used as the most popular mathematical survival model for recurrent event data (pregnancies are recurrent events). In cox model, the effect of predictors on the time between events could be determined by a hazard ratio (HR). The chi-square test was used to compare some characteristics between wives and husbands. For model building, first we fitted bivariable Cox regression between each predictor and outcome, then the predictor that has a *P*-value less than 0.15 was chosen to be further adjusted in assessing the effect of other covariates (bivariable *p*-values are not reported). We then created a multivariable Cox regression model including all predictors that have p-value < 0.15 in bivariable models. The adjusted hazard ratios (AHR) and 95% CIs were reported. Missing values were estimated from multiple imputations using a chained equation (MICE) algorithm under the assumption of a missing at random (MAR) mechanism [13]. The statistical software programs used for modeling were R version 4.3.3, package survival) and SPSS (version 20).

## Result

The mean age of women was 29.13 ± 3.2 years old. The mean Table 1 shows the descriptive characteristics of wives and husbands participated in the study. The result of chi-square test shows that the wives had significantly higher education level than the husbands (*P* < 0.001). Husbands used drugs significantly more than wives (P < 0.001). More than half of the families earned sufficient income (54.3%). The majority of couples had more than four times of intercourse each month (77.48%). The majority of wives (60.6%) got married over the age of 20 years old and most of them did not have any underlying disease (BP, Diabetes, kidney disease) (75.48%). The average of pregnancy spacing was 46 ± 1.2 month.

**Table 1:** Descriptive characteristic

Predictors	levels	*N* (%)
Education status of mother	Illiterate	7(0.52)
	Primary and Middle school	187(13.85)
	High school	730(54.07)
	University education	426(31.56)
Education status of husband	Illiterate	15(1.12)
	Primary and Middle school	271(20.07)
	High school	679(50.29)
	University education	385(28.52)
Mother drug use	Yes	47(3.5)
	No	1303(96.5)
Husband drug use	Yes	208(15.41)
	No	1142(84.59)
Number of intercourse (month)	< 2	41(3.04)
	2–4	263(19.48)
	> 4	1046(77.48)
Income status	Sufficient	733(54.3)
	Insufficient	617(45.7)
Mother underlying diseases (BP, Diabetes, kidney disease)	Yes	331(24.52)
	No	1019(75.48)
Mode of delivery	vaginal	945(70)
	C-section	405(30)
Contraception use	Yes	1025(75.93)
	No	325(24.07)
night job of mother	Yes	231(17.11)
	No	1119(82.89)
night job of husband	Yes	601(44.52)
	No	749(55.48)
	Mean ± SD	
Age of mother at marriage (year)	22.18 ± 4.10	
Breast feeding duration of preceding child (month)	25.33 ± 2.23	
mother’s BMI	26.53 ± 56.23	
Age of firs menstrual (year)	13.03 ± 1.23	
Mean number of children	5.10 ± 0.16	

Table 2 shows the results of Cox regression. The sign of coefficient shows direction of predictor’s effect on pregnancy spacing. The HR shows the rate of each predictor’s effect on pregnancy spacing. As observed, Mothers who are one year older at the age of marriage are 2 times more likely to have shorter pregnancy spacing (*P* < 0.001), HR = 1.931, 95%CI = (1.63, 2.02). Increasing one unit in BMI of the mother increased pregnancy spacing 1.69 times (*P* = 0.012), HR = 1.687, 95% CI = (1.51, 2.86). Mothers who used contraception are 2.34 times more likely to have longer pregnancy spacing (*P* < 0.001), HR = 2.337, 95% CI = (1.23, 2.76). Mothers who breast-fed their preceding children were 1.79 times more likely to have longer pregnancy spacing (*P* < 0.001), HR = 1.798, 95% CI = (1.35, 2.02). Presence of abortion or stillbirth in preceding pregnancy reduced pregnancy spacing 1.83 times (*P* = 0.033), HR = 1.831, 95% CI = (1.27, 2.93). Family income sufficiency increased pregnancy spacing 2.046 times (*P* = 0.018), HR = 2.046, 95% CI = (1.61, 3.02). Educated husbands were 1.5 times more likely to have longer pregnancy spacing (*P* = 0.035), HR = 1.53, 95% CI = (1.33, 2.48). Mothers who preferred sons were 2.23 times more likely to have shorter pregnancy spacing (*P* = 0.023), HR = 2.23, 95% CI = (1.24, 2.81). Mothers who knew about the optimal pregnancy spacing duration were 1.5 times more likely to have longer pregnancy spacing (*P* = 0.021), HR = 1.564, 95% CI = (1.33, 2.48).

**Table 2:** The effects of predictors on pregnancy spacing

predictors	Coef (*β*)	HR	Se(β)	95% CI for HR	*p*-value
**Mother predictors**					
Contraception use	0.849	2.337	0.136	1.23–2.76	**< 0.001**
age of mother at marriage	−0.659	1.931	0.003	1.63–2.02	**< 0.001**
Breast feeding duration of previous child	0.587	1.798	0.147	1.35–2.02	**< 0.001**
BMI of mother	0.523	1.687	0.447	1.51–2.86	**0.012**
First menstrual age	− 0.001	0.995	0.013	0.31–1.24	0.710
Mother having job	0.023	1.023	0.064	0.23–1.74	0.713
Mother drug use	−0.012	1.012	0.108	0.26–1.94	0.895
Mother underlying diseases (BP, Diabetes, kidney disease)	−0.013	1.013	0.107	0.98–1.78	0.891
**Education**					
Primary and Middle school	−0.011	1.011	0.107	0.21–1.72	0.891
High school	−0.063	1.065	0.097	0.37–1.54	0.359
University education	−0.074	1.076	0.094	0.31–1.61	0.281
night job of mother	0.066	1.068	0.096	0.28–1.74	0.469
Mode of delivery (vaginal, C-section)	0.038	1.038	0.074	0.41–1.72	0.621
**Husband predictors**					
**Education**					
Primary and Middle school	−0.018	1.018	0.074	0.38–1.61	0.511
High school	−0.036	1.036	0.074	0.32–1.97	0.632
University education	0.421	1.523	0.357	1.33–2.48	0.035
Husband drug use	0.038	1.038	0.056	0.29–1.92	0.499
Night job of husband	0.012	1.012	0.107	0.24–1.89	0.751
**Family and Child predictors**					
Income sufficiency	0.716	2.046	0.039	1.61–3.02	**0.018**
Mother’s awareness of optimum pregnancy spacing	0.447	1.564	0.227	1.33–2.48	**0.021**
Sex preference of mother (boy prefer)	−0.802	2.231	0.432	1.24–2.81	**0.023**
Presence of abortion or still birth in preceding pregnancy	−0.604	1.831	0.448	1.27–2.93	**0.033**
Sex preference of husband (boy prefer)	−0.045	1.046	0.056	0.61–1.84	0.478

*Coef* Coefficient, *HR* Hazard Ratio, *Se* standard error, *CI* Confidence Interval, *P*-value < 0.05 was significant and is bold in the table.

**Fig. 1:**
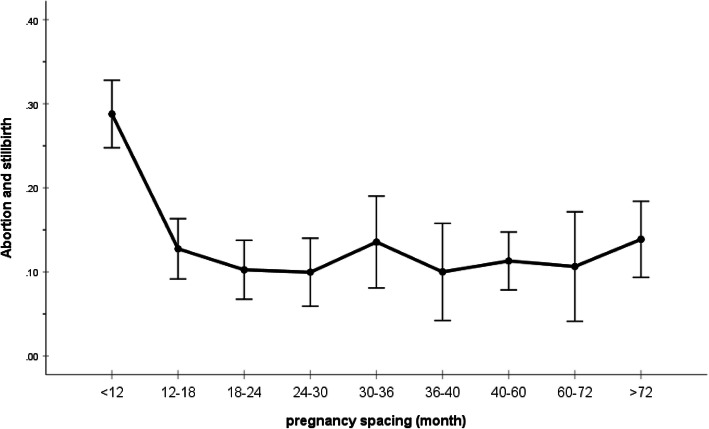
Comparison of the result of previous delivery and interval until next pregnancy

Figure 1 clarifies the effect of unsuccessful pregnancy (abortion or stillbirth) on decreasing pregnancy spacing. As observed, as the proportion of unsuccessful pregnancies increases the time until next pregnancy decreases.

## Discussion

The present study aimed to assess the associated factors of pregnancy spacing among women of reproductive age in the south of Iran. Factors affecting fertility rate and pregnancy spacing have been explored in previous studies [14, 15] but recurrent event analysis (survival analysis), which shows the effect of factors on repeatable events in a case, like pregnancy, has been rarely used. The results of the present study confirmed that the age of mother at first marriage, presence of stillbirth or abortion in preceding pregnancy, sex preference of mother after the preceding child (boy preference) are factors that decrease pregnancies spacing. In contrast, breast feeding practice, BMI of mother, contraception use, income sufficiency, father education, mother’s awareness of optimum pregnancy spacing are factors that increase pregnancies spacing. Woman age at marriage is found to have a significant correlation with shorter pregnancy spacing. This result was in accordance with the results of the previous studies that confirmed that woman who married at younger age have the chance of longer pregnancy spacing [16, 17]. This may be due to the fact that younger women have longer reproductive years and can manage their desired number of children with longer spaces. Presence of abortion or stillbirth in preceding pregnancy is found have a significant association with shorter pregnancy spacing. This result is in line with the result of previous studies [18, 19]. They may plan for having the next child sooner in order to forget previous sadness. The sex of the preceding child, especially son preference was strongly related to shorter pregnancy spacing. In a study conducted in India, there was a relation between son child preference and smaller pregnancy spacing [16]. There was another similar result that showed women with boy child in their first delivery, tended to have longer interval up to their next pregnancy [20]. Longer duration of breast feeding is found to have a significant relation with longer pregnancy spacing. This relation was found in a study conducted in Nigeria [21] and a similar study showed that women who breast-fed children more than 12 months became pregnant sooner compared to those who breast-fed them for 24 months or longer [22]. In the present study, mother’s BMI was found to be a significant predictor of pregnancy spacing. It was indicated that women with high BMI had longer pregnancy spacing than the ones with lower BMI. This may be due to the negative effect of obesity on fertility [23] that leads to longer duration between pregnancies. Obese women may want to have the next child sooner but have lower pregnancy chance. The findings of this study reveal that women who used contraception have longer pregnancy spacing. This result is in line with the results of study conducted in Ethiopia [22, 24] which showed that women who were not using contraceptives had shorter pregnancy spacing. This may be due to lack of ability to manage pregnancy intervals and they may have unwanted pregnancies. Contraception tools are helpful equipment to have optimum pregnancy spacing management [17]. The current study indicates that couples who were satisfied with their economic status tended to have longer pregnancy spacing. This result is in accordance with the result of the study that showed low income families tended to have more children with shorter pregnancy spacing [25]. This might be because the families with higher economic status usually had higher education status, too. Educated couples usually want fewer children; therefore, they manage to have their children with longer space if they have enough reproductive years. Husband’s education is found to be an influential factor in pregnancy spacing. This finding is consistent with that of another study in Iran which showed husbands with higher education tended to have longer pregnancy spacing [12]. This might be because educated husbands may have more information about the importance of pregnancy spacing for women and infants health. Lack of awareness about the advantages of optimal pregnancy spacing leads to decreasing pregnancy intervals. This result is similar to the result of the study that was conducted in Myanmar and showed that women who knew the benefit of optimal pregnancy spacing were found to have longer pregnancy spacing [26]. The awareness about optimal pregnancy spacing should be hightened by the government. Many wives and husbands do not have enough information about the benefits of managing pregnancy spacing. The drawbacks of short and long pregnancy spacing should receive more attention.

### Limitations

This study has some limitations. The effect of factors could be explored across a longitudinal study in order to show the significance of predictors with more precision. In the current study, women might have answered some questions according to their reminders; therefore, they may forget the accurate answers. The majority of predictors in this study were social-economic and behavioral factors. The role of more biological predictors could be explored in future studies.

## Conclusion

The most influential predictor of short and long pregnancy spacing are behavioral (contraception use, son preference of mother) and economical (income sufficiency). Therefor increasing individual knowledge and governments attempt in culture building and increasing economic status of families have essential role in the optimum pregnancy spacing.

## Supplementary information

**Additional file 1 Supplementary files 1.**English language version of checklist

**Additional file 2 Supplementary files 2** Persian language version of checklist

## Data Availability

The datasets used and/or analyzed during the current study are available from the corresponding author on reasonable request.

## Abbreviations

BMI: Body Mass Index
WHO: World Health Organization:
