# Supplementary material for: Associated factors of pregnancy spacing among women of reproductive age Group in South of Iran: cross-sectional study
Source: BMC Pregnancy Childbirth. 2020 Sep 22;20:554. doi: 10.1186/s12884-020-03250-x (PMC7510127; doi:10.1186/s12884-020-03250-x)
Supplement: Supplementary file 1 — Additional file 1 Supplementary files 1.English language version of checklist [file 12884_2020_3250_MOESM1_ESM.docx]

| **Reproductive history for married women aged 15-49** |
| --- |

| 1. | File Number: |
| --- | --- |
| 2. | Interviewer Name: |
| 3. | Observer Name: |
| 4. | Age of woman(year): |
| 5. | Date of marriage(year): |
| 6. | Education status of women:1- Illiterate 2- Primary and Middle school 3- High school 4- University education |
| 7. | Education status of husband: 1- Illiterate 2- Primary and Middle school 3- High school 4- University education |
| 8. | Age of firs menstrual (year) |
| 9. | How many times have you been pregnant? |
| 10. | Mother drug use: 1- Yes 2- No |
| 11. | Husband drug use: 11- Yes 2- No |
| 12. | Do you think your income is enough to live on? 1- Yes 2- No |
| 13. | History of Underlying disease of women disease: 1- Kidney disease 2- Diabetes 3- High blood pressure |
| 14. | Current menstrual status: 1- Regular 2- Irregular |
| 15. | Are you currently using contraception? 1- No 2- Yes |
| 16. | Average number of intercourse per month now: |
| 17. | Current weight of women(kg): |
| 18. | Height (cm): |
| 19. | Husband's date of birth(year): |
| 20. | night job of women: 1- Yes 2- No |
| 21. | night job of husband: 1- Yes 2- No |

**First pregnancy status:**

| 1. | Menstrual status before this pregnancy: 1- Regular 2- Irregular |
| --- | --- |
| 2. | Did you use contraception before this pregnancy? 1- No 2- Yes |
| 3. | Average frequency of intercourse in the month before this pregnancy: |
| 4. | Delivery result: 1- Live birth 2- Abortion 3- Still birth |
| 5. | Gender of baby: 1- Girl 2- Boy |
| 6. | Duration of breastfeeding (in months): |
| 7. | Sexual preference from the mother's point of view in this delivery: 1- Boy 2- Girl 3- It did not matter |
| 8. | Sexual preference according to the father in this delivery: 1- son 2- daughter 3- did not differ |
| 9. | Mode of delivery: 1- Vaginal 2- Cesarean section |

**Second pregnancy status:**

| 1. | Menstrual status before this pregnancy: 1- Regular 2- Irregular |
| --- | --- |
| 2. | Did you use contraception before this pregnancy? 1- No 2- Yes |
| 3. | Average frequency of intercourse in the month before this pregnancy: |
| 4. | Delivery result: 1- Live birth 2- Abortion 3- Still birth |
| 5. | Gender of baby: 1- Girl 2- Boy |
| 6. | Duration of breastfeeding (in months): |
| 7. | Sexual preference from the mother's point of view in this delivery: 1- Boy 2- Girl 3- It did not matter |
| 8. | Sexual preference according to the father in this delivery: 1- son 2- daughter 3- did not differ |
| 9. | Mode of delivery: 1- Vaginal 2- Cesarean section |

**Third pregnancy status:**

| 1. | Menstrual status before this pregnancy: 1- Regular 2- Irregular |
| --- | --- |
| 2. | Did you use contraception before this pregnancy? 1- No 2- Yes |
| 3. | Average frequency of intercourse in the month before this pregnancy: |
| 4. | Delivery result: 1- Live birth 2- Abortion 3- Still birth |
| 5. | Gender of baby: 1- Girl 2- Boy |
| 6. | Duration of breastfeeding (in months): |
| 7. | Sexual preference from the mother's point of view in this delivery: 1- Boy 2- Girl 3- It did not matter |
| 8. | Sexual preference according to the father in this delivery: 1- son 2- daughter 3- did not differ |
| 9. | Mode of delivery: 1- Vaginal 2- Cesarean section |

**Fourth pregnancy status:**

| 1. | Menstrual status before this pregnancy: 1- Regular 2- Irregular |
| --- | --- |
| 2. | Did you use contraception before this pregnancy? 1- No 2- Yes |
| 3. | Average frequency of intercourse in the month before this pregnancy: |
| 4. | Delivery result: 1- Live birth 2- Abortion 3- Still birth |
| 5. | Gender of baby: 1- Girl 2- Boy |
| 6. | Duration of breastfeeding (in months): |
| 7. | Sexual preference from the mother's point of view in this delivery: 1- Boy 2- Girl 3- It did not matter |
| 8. | Sexual preference according to the father in this delivery: 1- son 2- daughter 3- did not differ |
| 9. | Mode of delivery: 1- Vaginal 2- Cesarean section |

**Fifth pregnancy status:**

| 1. | Menstrual status before this pregnancy: 1- Regular 2- Irregular |
| --- | --- |
| 2. | Did you use contraception before this pregnancy? 1- No 2- Yes |
| 3. | Average frequency of intercourse in the month before this pregnancy: |
| 4. | Delivery result: 1- Live birth 2- Abortion 3- Still birth |
| 5. | Gender of baby: 1- Girl 2- Boy |
| 6. | Duration of breastfeeding (in months): |
| 7. | Sexual preference from the mother's point of view in this delivery: 1- Boy 2- Girl 3- It did not matter |
| 8. | Sexual preference according to the father in this delivery: 1- son 2- daughter 3- did not differ |
| 9. | Mode of delivery: 1- Vaginal 2- Cesarean section |
