# Supplementary material for: Associated factors of pregnancy spacing among women of reproductive age Group in South of Iran: cross-sectional study
Source: BMC Pregnancy Childbirth. 2020 Sep 22;20:554. doi: 10.1186/s12884-020-03250-x (PMC7510127; doi:10.1186/s12884-020-03250-x)
Supplement: Supplementary file 2 — Additional file 2 Supplementary files 2 Persian language version of checklist [file 12884_2020_3250_MOESM2_ESM.docx]

| **پرسشنامه تاريخچه باروري مخصوص زنان 15- 49 ساله ازدواج كرده** |
| --- |

| **1** | **شماره پرونده:** |
| --- | --- |
| **2** | **نام مصاحبه گر:** |
| **3** | **نام ناظر:** |
| **4** | **تاريخ تولد زن:** |
| **5** | **تاريخ ازدواج:** |
| **6** | **سواد زن: 1- بيسواد 2-ابتدائي 3- راهنمائي 4- دبيرستان** |
| **7** | **سواد شوهر: 1- بيسواد 2-ابتدائي 3- راهنمائي 4- دبيرستان** |
| **8** | **سن اولين قاعدگي:** |
| **9** | **تا به حال چند بار حامله شده ايد؟** |
| **10** | **مصرف موادمخدر زن در حال حاضر: 1- خير 2- بله** |
| **11** | **مصرف موادمخدر شوهر در حال حاضر: 1- خير 2- بله** |
| **12** | **آيا درآمدتان را براي زندگي كافي مي دانيد؟ 1- خير 2- بلي** |
| **13** | **سابقه بيماري مزمن: 1- - بيماري كليوي 2- بيماري قلبي 3- فشارخون بالا** |
| **14** | **وضعيت فعلي قاعدگي: 1- منظم 2- نامنظم** |
| **15** | **آيا در حال حاضر از وسائل يا روش پيشگيري از حاملگي استفاده مي كند؟ 1- خير 2- بلي** |
| **16** | **متوسط دفعات نزديكي در ماه در حال حاضر:** |
| **17** | **وزن فعلي ( كيلو گرم):** |
| **18** | **قد (سانتيمتر):** |
| **19** | **تاریخ تولد شوهر(سال)** |
| **20** | **شغل شب زن: 1-بله 2- خیر** |
| **21** | **شغل شب همسر: 1-بله 2- خیر** |

**وضعيت حاملگي اول:**

| **1** | **وضعيت قاعدگي قبل از اين حاملگي: 1- منظم 2- نامنظم** |
| --- | --- |
| **2** | **قبل از اين حاملگي از روشهاي پيشگيري از بارداري استفاده مي كرديد؟ 1- خير 2- بلي** |
| **3** | **متوسط دفعات نزديكي در ماه قبل از اين حاملگي:** |
| **4** | **نتيجه زايمان: 1- تولد زنده 2- سقط 3- مرده زائي** |
| **5** | **جنس نوزاد: 1- دختر 2- پسر** |
| **6** | **مدت شير دهي اين نوزاد (برحسب ماه):** |
| **7** | **ترجيح جنسي از نظر مادر در اين زايمان: 1- پسر 2- دختر 3- فرق نمي كرد** |
| **8** | **ترجيح جنسي از نظر پدر در اين زايمان: 1- پسر 2- دختر 3- فرق نمي كرد 4- نمي داند** |
| **9** | **نوع زايمان: 1- طبيعي 2- سزارين** |

**وضعيت حاملگي دوم:**

| **1** | **وضعيت قاعدگي قبل از اين حاملگي: 1- منظم 2- نامنظم** |
| --- | --- |
| **2** | **قبل از اين حاملگي از روشهاي پيشگيري از بارداري استفاده مي كرديد؟ 1- خير 2- بلي** |
| **3** | **متوسط دفعات نزديكي در ماه قبل از اين حاملگي:** |
| **4** | **نتيجه زايمان: 1- تولد زنده 2- سقط 3- مرده زائي** |
| **5** | **جنس نوزاد: 1- دختر 2- پسر** |
| **6** | **مدت شير دهي اين نوزاد (برحسب ماه):** |
| **7** | **ترجيح جنسي از نظر مادر در اين زايمان: 1- پسر 2- دختر 3- فرق نمي كرد** |
| **8** | **ترجيح جنسي از نظر پدر در اين زايمان: 1- پسر 2- دختر 3- فرق نمي كرد 4- نمي داند** |
| **9** | **نوع زايمان: 1- طبيعي 2- سزارين** |

**وضعيت حاملگي سوم:**

| **1** | **وضعيت قاعدگي قبل از اين حاملگي: 1- منظم 2- نامنظم** |
| --- | --- |
| **2** | **قبل از اين حاملگي از روشهاي پيشگيري از بارداري استفاده مي كرديد؟ 1- خير 2- بلي** |
| **3** | **متوسط دفعات نزديكي در ماه قبل از اين حاملگي:** |
| **4** | **نتيجه زايمان: 1- تولد زنده 2- سقط 3- مرده زائي** |
| **5** | **جنس نوزاد: 1- دختر 2- پسر** |
| **6** | **مدت شير دهي اين نوزاد (برحسب ماه):** |
| **7** | **ترجيح جنسي از نظر مادر در اين زايمان: 1- پسر 2- دختر 3- فرق نمي كرد** |
| **8** | **ترجيح جنسي از نظر پدر در اين زايمان: 1- پسر 2- دختر 3- فرق نمي كرد 4- نمي داند** |
| **9** | **نوع زايمان: 1- طبيعي 2- سزارين** |

**وضعيت حاملگي چهارم:**

| **1** | **وضعيت قاعدگي قبل از اين حاملگي: 1- منظم 2- نامنظم** |
| --- | --- |
| **2** | **قبل از اين حاملگي از روشهاي پيشگيري از بارداري استفاده مي كرديد؟ 1- خير 2- بلي** |
| **3** | **متوسط دفعات نزديكي در ماه قبل از اين حاملگي:** |
| **4** | **نتيجه زايمان: 1- تولد زنده 2- سقط 3- مرده زائي** |
| **5** | **جنس نوزاد: 1- دختر 2- پسر** |
| **6** | **مدت شير دهي اين نوزاد (برحسب ماه):** |
| **7** | **ترجيح جنسي از نظر مادر در اين زايمان: 1- پسر 2- دختر 3- فرق نمي كرد** |
| **8** | **ترجيح جنسي از نظر پدر در اين زايمان: 1- پسر 2- دختر 3- فرق نمي كرد 4- نمي داند** |
| **9** | **نوع زايمان: 1- طبيعي 2- سزارين** |

**وضعيت حاملگي پنجم:**

| **1** | **وضعيت قاعدگي قبل از اين حاملگي: 1- منظم 2- نامنظم** |
| --- | --- |
| **2** | **قبل از اين حاملگي از روشهاي پيشگيري از بارداري استفاده مي كرديد؟ 1- خير 2- بلي** |
| **3** | **متوسط دفعات نزديكي در ماه قبل از اين حاملگي:** |
| **4** | **نتيجه زايمان: 1- تولد زنده 2- سقط 3- مرده زائي** |
| **5** | **جنس نوزاد: 1- دختر 2- پسر** |
| **6** | **مدت شير دهي اين نوزاد (برحسب ماه):** |
| **7** | **ترجيح جنسي از نظر مادر در اين زايمان: 1- پسر 2- دختر 3- فرق نمي كرد** |
| **8** | **ترجيح جنسي از نظر پدر در اين زايمان: 1- پسر 2- دختر 3- فرق نمي كرد 4- نمي داند** |
| **9** | **نوع زايمان: 1- طبيعي 2- سزارين** |
